# New Dental Colleges

**Published:** 1868-10

**Authors:** 


*New Dental Colleges.*—Boston has now two Colleges of Dentistry which have received charters from the Legislature of the State of Massachusetts, under the names of "*The Boston Dental College,*" and the "*Dental School of Harvard University.*"

These new Colleges have been deemed necessary to meet a want long felt by the Dental profession of New England, as throughout these States no Dental College has heretofore existed; and both in their first annual Circulars profess their aim to be to raise the standard of dental education and professional excellence. We sincerely hope that their expectations may be fully realized, as the faculties of both these new institutions contain gentlemen well known in our profession; four of them are graduates of the Baltimore College of Dental Surgery.

With such a wide field as the New England States present, we think there is no reason to doubt the success of both these Colleges; and although considerable feeling of an unpleasant nature was manifested in the efforts to obtain charters, yet we hope that now both are fairly organized, the rivalry between them may be based on merit alone.

The faculty of the "Boston Dental College" consists of I. H. Wetherbee, D. D. S., President and Prof. ot Dental Science and Operative Dentistry. W. H. Atkinson, M. D., Prof. Hygiene and Dental Jurisprudence. A. Lawrence, D. D. S., Prof. Institutes of Dentistry. W. S. Miller, D. D. S., and C. G. Davis, D. D. S., Adjunct Profs. Dental Science and Operative Dentistry. S. J. McDougall, M. D., Prof. Dental Art and Mechanism. H. F. Bishop, D. D. S., Adjunct Prof. Dental Art and Mechanism. R. King Browne, M. D., Prof. Anatomy and Physiology. J. B. Ordway, M. D., Adjunct Prof. Anat. and Physiology. L. R. Sheldon, M. D., Prof. Pathology and Therapeutics. J. A. Follett, M. D., Dean, and Prof. Prin. and Prac. of Surgery. F. W. Clark, S. B., Prof. Chemistry and Metallurgy.

We observe the name of R. King Browne, M. D. in the Ohio Dental College Faculty also: some misunderstanding doubtless.

The "Dental School of Harvard" evidently considering the title of "Doctor of Dental Surgery" not quite dignified enough for a department of Harvard University, have determined to confer the degree of Doctor of Dental Science instead. It is fortunate for the community at large that the initials D. D. S. are the same for both titles. The faculty of Harvard Dental School consists of N. C. Keep, M. D., D. D. S., Dean and Prof. Mechanical Dentistry. O. W. Holmes, M. D., Prof. Anatomy and Physiology. H. J. Bigelow, M.D. Prof. Surgery. J. Bacon, M. D., Prof. Chemistry. T. B. Hitchcock, M. D., Prof. Dental Pathology and Therapeutics. G.T. Moffatt, M. D., Prof. Operative Dentistry. L. D. Shepard, D. D. S., Adjunct Prof. Operative Dentistry. J. A. Salmon, D. D. S., Lecturer on Operative Dentistry. N. W. Hawes, Demonstrator of Operative Dentistry. S. T. Ham, Demonstrator Mechan. Dentistry. C. B. Porter, M. D., Demonstrator of Anatomy.

The Boston Dental College has joined the Association of Dental Colleges.

